# Seasonality of MRSA Infections

**DOI:** 10.1371/journal.pone.0017925

**Published:** 2011-03-23

**Authors:** Leonard A. Mermel, Jason T. Machan, Stephen Parenteau

**Affiliations:** 1 Department of Epidemiology and Infection Control, Rhode Island Hospital, Providence, Rhode Island, United States of America; 2 Department of Medicine, Warren Alpert Medical School of Brown University, Providence, Rhode Island, United States of America; 3 Department of Orthopaedics, Warren Alpert Medical School of Brown University, Providence, Rhode Island, United States of America; 4 Department of Surgery, Warren Alpert Medical School of Brown University, Providence, Rhode Island, United States of America; National Institute of Allergy and Infectious Diseases, National Institutes of Health, United States of America

## Abstract

Using MRSA isolates submitted to our hospital microbiology laboratory January 2001–March 2010 and the number of our emergency department (ED) visits, quarterly community-associated (CA) and hospital-associated (HA) MRSA infections were modeled using Poisson regressions. For pediatric patients, approximately 1.85x (95% CI 1.45x–2.36x, adj. p<0.0001) as many CA-MRSA infections per ED visit occurred in the second two quarters as occurred in the first two quarters. For adult patients, 1.14x (95% CI 1.01x–1.29x, adj.p = 0.03) as many infections per ED visit occurred in the second two quarters as in the first two quarters. Approximately 2.94x (95% CI 1.39x–6.21x, adj.p = 0.015) as many HA-MRSA infections per hospital admission occurred in the second two quarters as occurred in the first two quarters for pediatric patients. No seasonal variation was observed among adult HA-MRSA infections per hospital admission. We demonstrated seasonality of MRSA infections and provide a summary table of similar observations in other studies.

## Introduction

Seasonal variation in *Staphylococcus aureus* infections is controversial. Some studies have demonstrated a highly significant surge of such infections during the warmest months and during autumn in temperate and tropical settings [1]–[3]. However, a recent study did not find such a correlation [4]. We have observed peaks of community-associated methicillin-resistant *S. aureus* (CA-MRSA) infections during the summer and autumn and we set out to determine if there is a correlation between MRSA infections in patients of all ages and seasonality.

## Results

**Figure 1:**
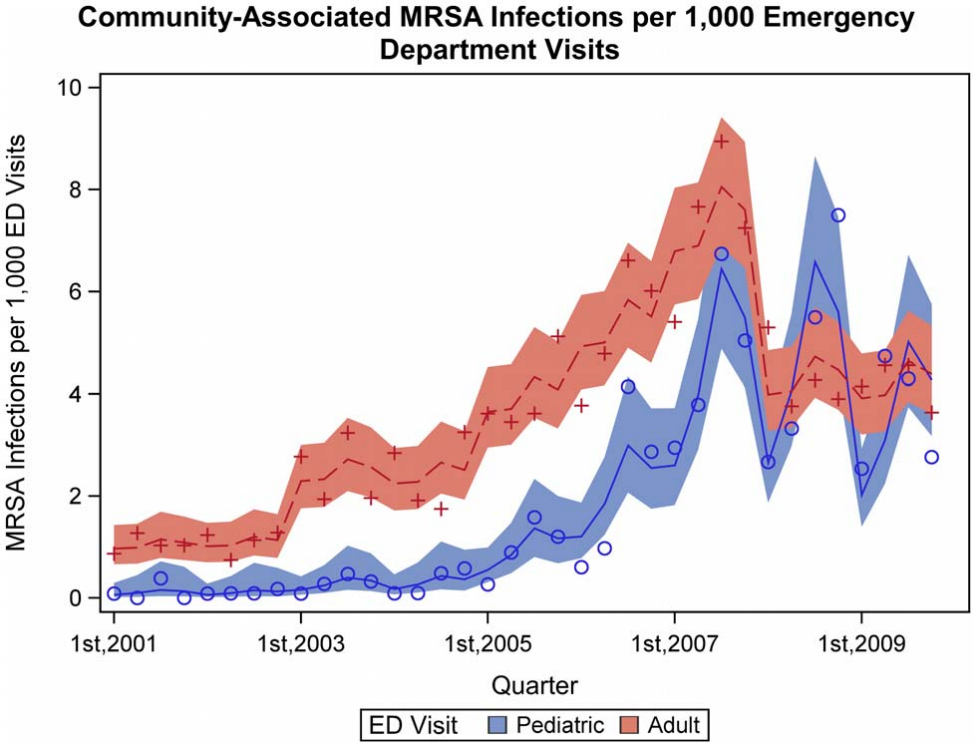
Community-associated MRSA infections along with the model predictions and 95% confidence intervals.

**Figure 2:**
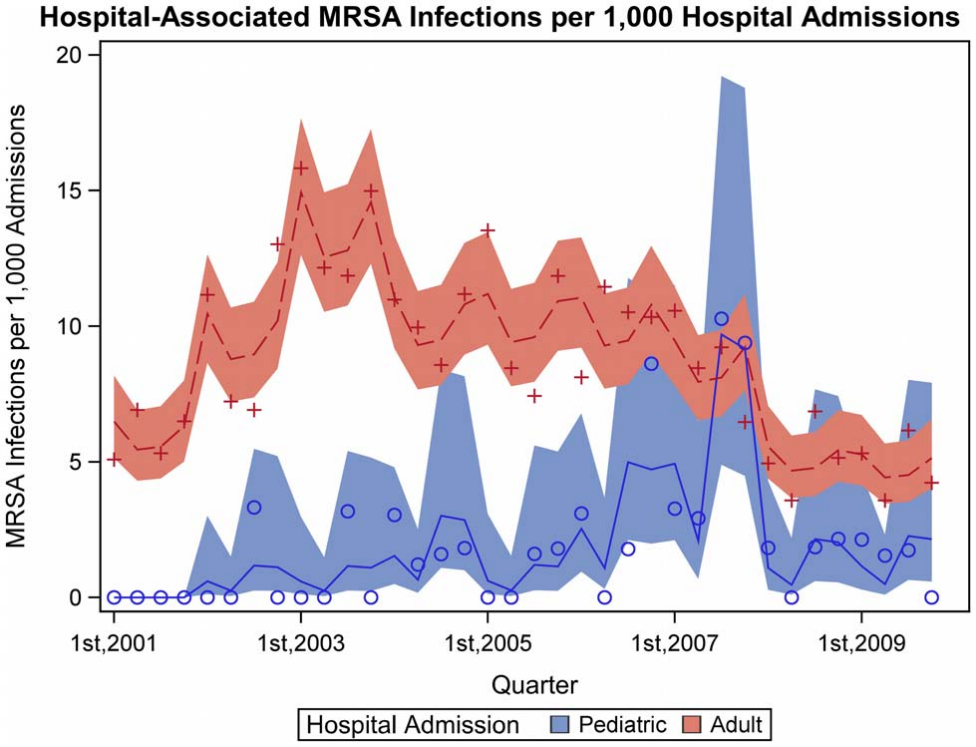
Hospital-associated MRSA infections along with the model predictions and 95% confidence intervals.

We found strong evidence of a seasonal effect in the rate of CA-MRSA isolates per ED visit in both pediatric and adult patients (Figure 1). For pediatric patients, a mean of approximately 1.85x (95% CI 1.45x–2.36x, adj. p<0.0001) as many CA-MRSA infections per ED visit occurred in the second two quarters as occurred in the first two quarters over the study period. For adult patients, the effect per number of ED visits was smaller (adj.p = 0.001), which indicated 1.14x (95% CI 1.01x–1.29x, adj.p = 0.03) as many infections per ED visit in the second two quarters as in the first two quarters. Hospital-associated MRSA (HA-MRSA) infections in pediatric patients had seasonal variation (Figure 2). A mean of approximately 2.94x (95% CI 1.39x–6.21x, adj.p = 0.015) as many HA-MRSA infections per hospital admission occurred in the second two quarters as occurred in the first two quarters over the study period. This reflected greater seasonal variation than we observed among adult HA-MRSA infections per hospital admission (adj.p = 0.011), in which the second two quarters did not differ from the first two quarters, 1.00x (95% CI 0.89x–1.12x, adj.p = 0.97).

## Discussion

Seasonality of staphylococcal infections is not noted in many textbook chapters or review articles. Nevertheless, several investigators have demonstrated seasonal variation in *S. aureus* infections, particularly skin infections, with a preponderance of infections during the summer and autumn (Tables 1 and 2). As noted in Tables 1 and 2, seasonal variability has been observed in pediatric and adult patients in temperate and tropical climates. We found a two to three-fold increased incidence of MRSA infections in pediatric patients during the second two quarters over the last decade. A lesser degree of seasonal variation was observed in adult CA-MRSA infections. It is difficult to explain the seasonality of pediatric HA-MRSA infections; however, we suspect that many of these patients were colonized with MRSA at the time of hospitalization and infections developed while hospitalized. Since 90% of pediatric hospital visits in Rhode Island are at our hospital, our data represents the vast majority of pediatric MRSA infections seen at local hospitals.

**Table 1:** Seasonality and *Staphylococcus aureus* Mixed Infections, Bacteremia, and Upper Extremity Infections.

Type of Infection	Age	Locale	Seasonality	Author
CA*-MRSA† (USA 300 & USA 400 strains) infections	All	Iowa	Peak incidence: Summer (47% of cases occurred Jun 21-Sep 22; p<0·001)	[1]
CA-MRSA severe infections	All	Georgia	Peak incidence: Summer (∼34% of casesoccurred Jul-Sep; ∼45% occurred Jul-Oct)	[5]
CA-MRSA infections in military recruits	Adult	Georgia	Peak incidence: Summer (∼ 41% of cases occurred Jul-Sep; ∼54% of cases occurred Jul-Oct)	[6]
CA- and HA‡-*S. aureus* infections	All	Scotland	Peak incidence: ‘Spring seasonal variation of MRSA’was observed but not for MSSA§	[7]
CA- and HA-*S. aureus* infections requiring hospitalization	Adult	Maryland	Peak incidence: Jul-Sep (IRR 1·04; p = 0·5)	[4]
CA-*S. aureus* infections	Pediatric	Texas	Monthly peak incidence: 2002 = Jul; 2003 = Aug; 2004 = May	[8]
CA-*S. aureus* infections	Pediatric	Greece	Peak incidence: Summer (72% of cases occurred Jul-Sep)	[9]
CA-*S. aureus* bacteremia	All	Connecticut	Peak incidence: Jul-Sep (RR 1·0; p = non-significant)	[10]
CA-*S. aureus* bacteremia	Adult	Taiwan	Peak incidence: ‘Late autumn and early winter’(26% of cases occurred Jul-Sep; 42% of cases occurred Jul-Oct)	[11]
*S. aureus* bacteremia	All	Massachusetts	Peak incidence: ‘There was no peak of incidence atany season of the year’	[12]
*S. aureus* pneumonia and secondary bacteremia	All	Maryland	Peak incidence: Summer and winter (30% of cases occurred May-June; 30% of cases occurred Nov-Dec)	[13]
Upper extremity (PVL gene positive) *S. aureus* infections	All	Greece	Peak incidence: Summer (56% of cases occurred Jun-Aug)	[14]

*CA  =  Community-associated;

† MRSA  =  Methicillin-resistant *S. aureus*;

‡ HA  =  Hospital-associated;

§ MSSA  =  Methicillin-susceptible *S. aureus.*

**Table 2:** Seasonality and *Staphylococcus aureus* Skin Infections.

Type of Infection	Age	Locale	Seasonality	Author
CA*-associated *S. aureus* ‘boil infections’	All	Nigeria	Peak incidence: 33% of cases occurred during warmest recorded months (Jan–Mar)	[15]
CA-associated pyoderma	All	India	Peak incidence: Summer (40% of cases occurred Jun–Aug)	[16]
CA-associated pyoderma	All	Malawi	Peak incidence: Summer (Dec–Apr)	[17]
CA-associated pyoderma	Pediatric	India	Peak incidence: 68% of cases ‘reported during the hot and humid months of Jun–Sep'	[18]
Dermatitis cruris pustulosis exacerbation (87% culture-positive for *S. aureus*)	All	India	Peak incidence: Summer (87% of cases)	[19]
Impetigo	Pediatric	Nether-lands	Peak incidence in 1987 & 2001: Summer (‘incidence was significantly higher in summer’)	[20]
Impetigo	Pediatric	United Kingdom	Peak incidence: ‘Late Summer’ (∼37% of cases Jul–Sep; seasonal effect [p = 0·02]; correlation between impetigo andmean temperature the previous month [r = 0·55; p = 0·001])	[3]
Impetigo	Pediatric	United Kingdom	Peak incidence: Autumn (Oct peak in 4 of 5 years studied); ∼1–2 months after the month with the highest average temperature	[21]
Impetigo	Pediatric	Alabama	Peak Incidence: Summer (33% of cases occurred in Aug; monitored Jul–Jan rather than the calendar year)	[22]
Impetigo	Pediatric	Australia	Peak incidence: 79% of cases occurred in summer and autumn	[23]
Impetigo	Pediatric	Pakistan	Peak incidence: Summer (2–3 fold increased incidence/100 person-wks of impetigo in Jul compared with May, Sep, or Oct)	[24]
Impetigo bullosa due to fusidic acid-resistant *S. aureus*	Pediatric	Norway	Peak incidence: ‘Marked seasonal fluctuation with the highest prevalence in early autumn’ (52% of 2001 cases in Aug)	[25]

*CA  =  Community-associated.

We believe that it is the sequence of 3^rd^ and 4^th^ quarters which is important in demonstrating the peak in MRSA infections in temperate regions rather than just the warmest quarter of the year. We reviewed meteorologic data for Rhode Island during the decade of our study period and found that the 2^nd^ quarter was warmer, on average, than the 4^th^ quarter. Thus, we believe that it is the sequence of 3^rd^ and 4^th^ quarters that is important and not the 3^rd^ quarter in isolation. We previously demonstrated transmission of an identical MRSA pulsotype in a large extended family living in different homes over 2–4 weeks [26]. One study found a correlation between impetigo and the mean ambient temperature during the previous month [r = 0·55; p = 0·001]; 3). Another study demonstrated a 1–2 month lag between peak temperature and peak incidence of impetigo in children and this was ascribed to more insect bites during peak temperatures [21]. Thus, an increased incidence of infection in autumn (4^th^ quarter), may reflect a lag between Staphylococcal colonization and subsequent infection. Additionally, an increased incidence of viral respiratory infections in autumn, possibly reflecting transmission in more confined settings such as schools, may add to greater *S. aureus* transmission and subsequent infection during this time [27]. Hydration of the stratum corneum of the skin is an important in promoting microbial growth [28]. Hydration would be maximized when high temperatures promote sweat production and in high relative humidity. The presence of both factors may be critically important in providing the environmental conditions that facilitate heavy growth of *S. aureus* on skin [16]. Nevertheless, one investigator found an inverse correlation between humidity and S. aureus skin infections [15]. Some investigators did not observe any significant seasonality of *S. aureus* infections [4], [10], [12]. Since many reports of seasonality of *S. aureus* infections involve skin infections, it may be that these investigators had fewer such infections in their patient population thus masking the effect.

Grassley and Fraser [29] describe variables that explain seasonality of infectious diseases: pathogen survival outside the host; host behavior; host immune function; and abundance of vectors and non-human hosts. There is a correlation between the quantity of *S. aureus* in the nares and on clothing [30] and a correlation between the quantity of *S. aureus* in the nares, on skin, and the quantity of *S. aureus* in the surrounding air [30]–[32]. Increased *S. aureus* transmission during the summer months may reflect a greater density of this microbe on the skin, nares, throat, and/or perineum during warmer, and often more humid conditions [28], it may reflect other environmental conditions conducive to disease spread, or both. Some authors have not observed seasonal variation in *S. aureus* nasopharyngeal carriage [33]–[35] but these studies may have been underpowered to find such a difference and they did not assess colonization of other body sites.

It is hoped that the current study will prompt further investigation into the seasonality of *S. aureus* infections to better understand the biologic basis for this observation.

## Methods

We collected microbiologic data on all clinical (i.e., non-surveillance) MRSA isolates submitted to our hospital microbiology laboratory between January 2001 and March 2010. MRSA isolates were identified using standard microbiologic methods. We also determined the number of visits to our adult and pediatric emergency departments (ED) during this time. We defined CA-MRSA as isolates obtained within 48 hours of hospitalization and HA-MRSA based on isolates obtained after 48 hours of hospitalization, or for surgical wound infections, those who had their procedure within the prior 30 days.

Quarterly CA-MRSA and HA-MRSA infection counts were modeled using Poisson regressions, offset with the log number of ED visits or log number of admissions with lengths of stay of three or more days, respectively, to adjust for changes in the number of patients seen. For CA-MRSA infections, the log number of ED visits for each quarter was used as an offset variable, making the prediction rates per ED visit. Models included effects for ED visits (pediatric vs. adult), categorical year, categorical quarter, and the interactions between ED and year, and ED and quarter. For HA-MRSA infections, the log number of hospital admissions with lengths of stay of three or more days for each quarter was used as an offset variable, making the prediction rates hospital admission. Models included effects for hospital admission of three or more days (pediatric vs. adult), categorical year, categorical quarter, and the interactions between hospital admission and year, and hospital admission and quarter. Models and hypothesis tests were adjusted for slight overdispersion based on the Pearson Chi-Square. Complex comparisons were used to test whether quarters 1 and 2 were significantly different from quarters 3 and 4 within each (i.e., adult or pediatric) ED visit or hospital admission and to test for a difference between ED visit or hospital admission in the size of these effects. Alpha was maintained at 0.05 across all three hypothesis tests using the Holm test (alpha per comparison  =  0.0167). This study was approved by the Rhode Island Hospital Institutional Review Board. Informed consent was not obtained as patient-specific data is not reported and data had already been abstracted in our infection control software program.
